# Multilevel analysis quantifies variation in the experimental effect while optimizing power and preventing false positives

**DOI:** 10.1186/s12868-015-0228-5

**Published:** 2015-12-19

**Authors:** Emmeke Aarts, Conor V. Dolan, Matthijs Verhage, Sophie van der Sluis

**Affiliations:** Department of Functional Genomics, Center for Neurogenomics and Cognitive Research, VU University Amsterdam, De Boelelaan 1085, 1081 HV Amsterdam, The Netherlands; Department of Molecular Computational Biology, Max Planck Institute of Molecular Genetics, Ihnestraße 63-73, 14195 Berlin, Germany; Department of Biological Psychology, VU University Amsterdam, Van der Boechorststraat 1, 1081 BT Amsterdam, The Netherlands; Department of Clinical Genetics, Section Functional Genomics, VU Medical Center Amsterdam, De Boelelaan 1085, 1081 HV Amsterdam, The Netherlands; Department of Clinical Genetics, Section Complex Trait Genetics, VU Medical Center, De Boelelaan 1085, 1081 HV Amsterdam, The Netherlands

**Keywords:** Multilevel analysis, False positive rate, Pseudo-replication, Statistical power, Hierarchical data, Clustered data, Optimal research design, Experimental effect, Neuroscience

## Abstract

**Background:**

In neuroscience, experimental designs in which multiple measurements are collected in the same research object or treatment facility are common. Such designs result in clustered or nested data. When clusters include measurements from different experimental conditions, both the mean of the dependent variable and the effect of the experimental manipulation may vary over clusters. In practice, this type of cluster-related variation is often overlooked. Not accommodating cluster-related variation can result in inferential errors concerning the overall experimental effect.

**Results:**

The exact effect of ignoring the clustered nature of the data depends on the effect of clustering. Using simulation studies we show that cluster-related variation in the experimental effect, if ignored, results in a false positive rate (i.e., Type I error rate) that is appreciably higher (up to ~20–~50 %) than the chosen $\alpha$-level (e.g., $\alpha$ = 0.05). If the effect of clustering is limited to the intercept, the failure to accommodate clustering can result in a loss of statistical power to detect the overall experimental effect. This effect is most pronounced when both the magnitude of the experimental effect and the sample size are small (e.g., ~25 % less power given an experimental effect with effect size *d* of 0.20, and a sample size of 10 clusters and 5 observations per experimental condition per cluster).

**Conclusions:**

When data is collected from a research design in which observations from the same cluster are obtained in different experimental conditions, multilevel analysis should be used to analyze the data. The use of multilevel analysis not only ensures correct statistical interpretation of the overall experimental effect, but also provides a valuable test of the generalizability of the experimental effect over (intrinsically) varying settings, and a means to reveal the cause of cluster-related variation in experimental effect.

**Electronic supplementary material:**

The online version of this article (doi:10.1186/s12868-015-0228-5) contains supplementary material, which is available to authorized users.

## Background

**Fig. 1:**
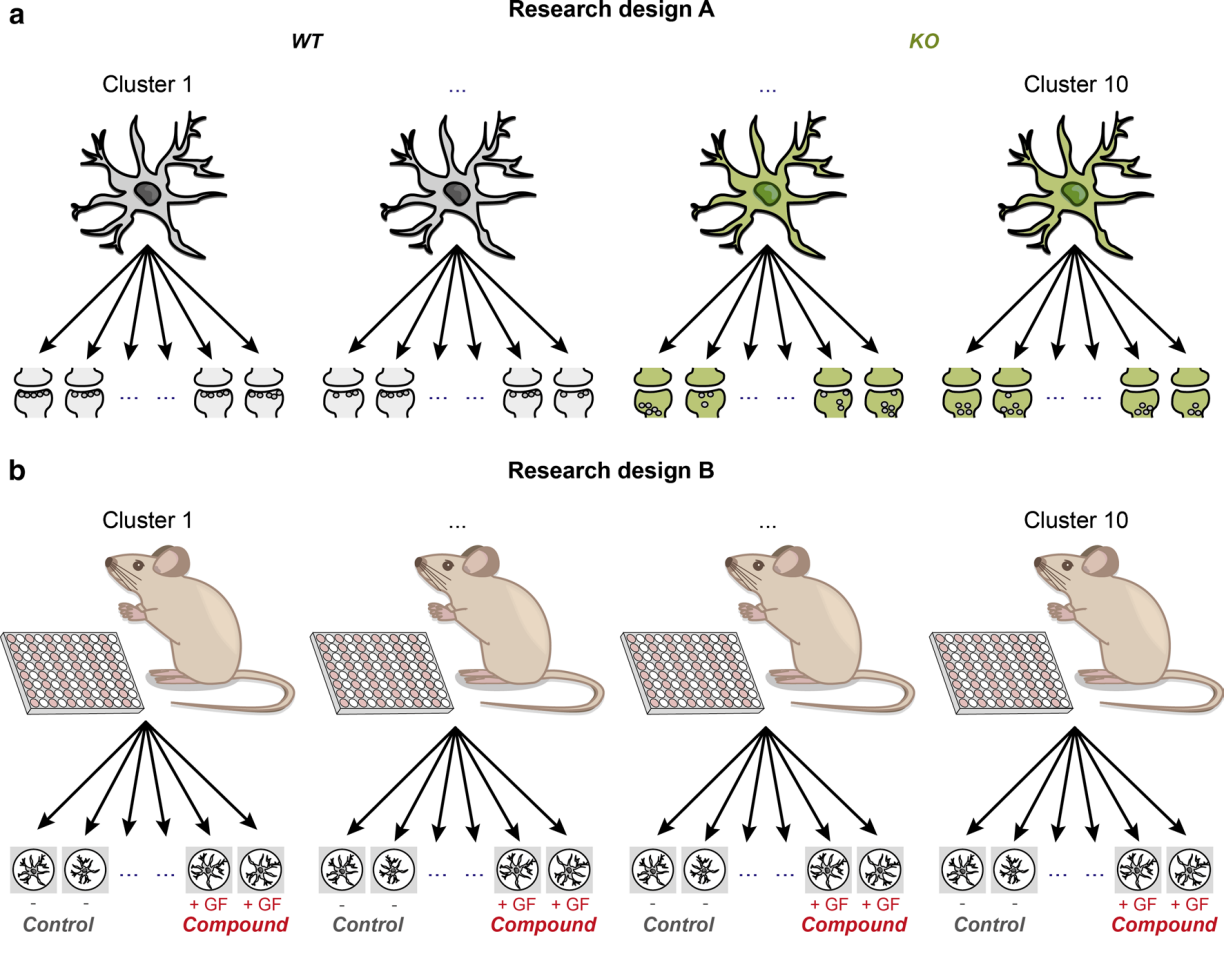
Graphical illustration of nested data in research design A and B. In design A **a**, all observations in a cluster are subject to the same experimental condition. An example of this design is the comparison of WT and KO animals with respect to the number of docked vesicles within presynaptic boutons: bouton-measurements are typically clustered within neurons, and all measurements from the same neuron belong to the same experimental condition, i.e., have the same genotype. In this hypothetical example, we assume that a single neuron is sampled from each animal. If multiple neurons are sampled from the same animal, a third “mouse” level is added to the nested structure of the data. In research design B **b**, observations from the same cluster are subject to different experimental conditions. An example of this design is the comparison of neurite outgrowth in cells that are treated, or not (control), with growth factor (GF). Here, typically multiple observations from both treated and untreated neurons are obtained from, and so clustered within, the same animal

Nested data are common in neuroscience, where multiple observations are often collected in the same cell, tissue sample, litter, or treatment facility [1–4]. For example, consider a study of differences between wild type (WT) and knock-out (KO) animals in the number of docked vesicles within presynaptic boutons. As each neuron has multiple presynaptic boutons, one can measure the number of docked vesicles in multiple boutons of every neuron, resulting in multiple measurements within each neuron (Fig. 1a). As the measurements are clustered within neurons, data resulting from this type of experimental design is referred to as clustered or nested data.^1^ Such data have a hierarchical, or multilevel, structure. In the present example, the number of presynaptic boutons within a neuron is referred to as the level 1 variable, and neuron is the level 2, clustering, variable. In this research design, which we refer to as design A, all observations from the same cluster belong to the same experimental condition (in our example: genotype). Research design A has received considerable attention in neuroscience literature, emphasizing that such clustered data are common in neuroscience, and that statistical accommodation of the clustered nature of the data is crucial to avoid false positive results (i.e., inflation of the Type I error rate) [1–6].

Nested data, however, may arise in designs other than design A. In what we call research design B, observations from the *same* cluster are subjected to different experimental conditions. Classical examples are studies in which mice from the same litter are randomized over different experimental treatments. Research design B is common in the clinical and preclinical neurosciences [2, 7], but is also employed in the basic neurosciences. Examples include studies on the effect of different pharmacological compounds, recombinant proteins, or siRNA’s on cellular or subcellular features, where the experimental treatment is applied to different tissue samples of the same animal (Fig. 1b). Other examples include the comparison of morphological features from animals or tissue samples, where each animal or tissue sample provides multiple measurements on different morphological features. Examples of research design B data in biological neuroscience are given in Table 1.

**Table 1:** Examples of research design B nested data in biological neuroscience

Example	Individual observations from	Clustered in	Experimental variable	Experimental effect
Effect of a growth factor on neurite outgrowth	Neurons	Animals	Growth factor yes/no	Difference in neurite outgrowth
Effect of neurite location (axon/dendrite) on traveling speed of intracellular vesicles	Intracellular vesicles	Neurons	Axon or dendrite	Difference in travelling speed
Effect of neuron type (interneurons/projection neurons) on electrophysiological properties	Neurons	Animals	Neuron type	Difference in electrophysiological properties
Effect of spine morphological features on synapse compartmentalization	Spines	Neurons	Morphological type	Difference in synapse compartmentalization

In neuroscience literature, the discussion of research design B has been limited to the case in which the experimental effect is assumed to be the same for all clusters [2, 4]. This is a strong assumption, and there is often no reason to believe that the experimental manipulation will indeed have exactly the same effect in each cluster. Here we show that even a small amount of variation in the experimental effect across clusters inflates the false positive rate of the experimental effect, if that variation is not accommodated in the statistical model.

The aim of the present paper is to describe the intricacies of research design B, and explain how these can be accommodated in multilevel analysis (also known as ‘hierarchical modeling’, ‘mixed-’ or ‘random effects models’). In Neuroscience, the research question in nested designs is often formulated at the level of the individual observations. However, as a result of the clustering, the individual observations may show dependency, and this dependency needs to be accommodated in the statistical analysis. First, we briefly discuss research design A. Second, we focus specifically on the defining features of research design B, and show how these can be accommodated in multilevel analysis. Third, we demonstrate through simulations that misspecification of the statistical model for data obtained in design B results either in increased Type I error rate (i.e., spurious effects), or in decreased statistical power to detect the experimental effects. Finally, we discuss the use of cluster-related information to explain part of the variation in the experimental effect, with the aim of increasing statistical power to detect the experimental effect, and facilitating the biological understanding of variation in this effect.

### Research design A

In research design A, multiple observations are collected in the same cluster, and only one experimental condition is represented in each cluster (Fig. 1a). We recently emphasized that design A is common in neuroscience research: at least 53 % of research papers published in 5 high profile neuroscience journals concerned data collected in this design [1]. This design has received some attention in the neuroscience literature, focusing specifically on ways to correctly analyze such data [1–4]. Our central message was that multiple measurements per cluster (e.g., neuron or mouse) cannot be considered independent observations, since measurements from the same cluster tend to be more similar to each other than to measures from different clusters. This can result in systematic differences between clusters, i.e., the mean of the dependent variable varies across clusters. Clustering implies that this variation exceeds that arising from random sampling fluctuation of individual observations within a cluster^2^ (i.e., within cluster variation). Standard statistical techniques, such as regression analysis, *t* test, and ANOVA are unsuited to analyze clustered data, because these techniques rely on the assumption that all observations are independent. Given dependency, they produce underestimated standard errors, and so underestimated p values. The result is (possibly considerable) inflation of the Type I error rate, i.e., false positive rate (see [1] for an explanation on, and estimates of, this inflation).

There are two ways to handle research design A data. One can average across all observations within each cluster and apply standard techniques using these means, which are independent observations. Alternatively, avoiding the data reduction associated with such averaging, a multilevel model can be used to accommodate the nested structure. In multilevel analysis, the comparison of experimental conditions is conducted on the cluster level means, while retaining the distinction between the variance within clusters (e.g., differences between observations within a mouse) and variance between clusters (e.g., differences between the mice in cluster level means). See “Box 1” for a description of the statistical multilevel model for design A. Of these two approaches, multilevel analysis is preferable as it exploits all available information, and confers the greatest statistical power [1, 4]. The multilevel model also allows one to obtain the intracluster correlation (ICC), which quantifies the degree to which measurements from the same cluster are more similar to each other than to measures from different clusters. The ICC ranges between 0 (there is no variation between clusters and thus no dependency) and 1 (observations within clusters are equivalent and observations over clusters are different: complete dependency, i.e., the value of the observations depends completely on cluster-membership; see “Box 1”). The ICC is the standardized version of the variance between clusters, denoted by $\sigma_{u0}^{2}$, and also referred to as the intercept variance (i.e., the variance in the cluster level means).

## Box 1: Multilevel model for research design A

In the multilevel model for research design A, the nested structure of the data is accommodated by specifically incorporating the variation in cluster means, i.e., in the intercepts, in the statistical model. In case of a 2-level multilevel model, we have a level 1 model, the model of the individual observations, and a level 2 model, the cluster level model. The level 1 model takes on the following form:

1 $$Y_{ij} = \beta_{0j} + e_{ij} \quad {\text{with }} \quad e_{ij} \sim N(0,\sigma_{e}^{2} ),$$

i.e., the dependent variable *Y* for observation *i* from cluster *j* is predicted from the cluster *j* specific mean value of Y in cluster *j,* denoted by the cluster-specific intercept $\beta_{0j}$, and the zero mean residual $e_{ij}$. The variation in the intercept is specifically modeled in the cluster level model. Without incorporating an experimental effect of a cluster level experimental manipulation, the cluster level model is:

2 $$\beta_{0j} = \gamma_{00} + u_{0j} \quad {\text{with }} \quad u_{0j} \sim N(0,\sigma_{u0}^{2} ),$$

where $\gamma_{00}$ is the overall mean value of Y calculated across all clusters, and $u_{0j}$ is the cluster *j* specific deviation from that overall mean value. Hence, a distinction is made between ‘between cluster variation’, $\sigma_{u0}^{2}$ (i.e., the variance of $u_{0j}$), and the remaining within clusters variation, $\sigma_{e}^{2}$ (i.e., the variance of $e_{ij}$). Greater variation between clusters corresponds to a higher relative similarity of observations from the same cluster. Therefore, a standardized measure for dependency is given by the intracluster correlation (ICC), which represents the degree of relative similarity of observations from the same cluster and is obtained by:

3 $$ICC = \frac{{\sigma_{u0}^{2} }}{{\sigma_{u0}^{2} + \sigma_{e}^{2} }},$$

i.e., the variance between clusters divided by the total variance in the data. This ICC ranges between 0 (there is no variation over clusters and thus no dependency) and 1 (observations within clusters are equivalent and observations over clusters are different: complete dependency, i.e., the value of the observations depends completely on cluster-membership).

Commonly, designs in neuroscience involve two conditions, e.g., a control and an experimental condition. To extend the model to include the effect of an experimental manipulation at the cluster level (i.e., difference between the experimental and control condition), and (partly) explain the differences in mean between clusters (i.e., different intercepts), we expand the cluster level model in Eq. 2 as follows:

4 $$\beta_{0j} = \gamma_{00} + \gamma_{01} *Z_{j} + u_{0j} \,\, {\text{with }} \,\, u_{0j} \sim N(0,\sigma_{u0}^{2} ),$$

where $Z_{j}$ is a (cluster level) dummy coded indicator variable that denotes the experimental condition of cluster *j* (e.g., 0 for WT and 1 for KO), $\gamma_{00}$ is the overall intercept, that denotes the overall mean in the control condition given that the indicator variable *Z*_*j*_ equals 0 for the control condition, $\gamma_{01}$ is the overall deviation of the experimental condition from the control condition given that the indicator variable *Z*_*j*_ equals 1 for the experimental condition (i.e., from the intercept $\gamma_{00}$), and $u_{0j}$ is the cluster specific deviation from the overall intercept that remains after taking the effect of the experimental condition into account. Note that if $\sigma_{u0}^{2}$ = 0, clustering is effectively absent, which renders the subscript *j* superfluous. In that specific case, the model at the level of the individual observations reduces to $Y_{i} = \gamma_{00} + \gamma_{01} *Z + \text{ }e_{i}$, i.e., the standard *t* test written in regression terms.

Having discussed research design A, we now present the defining features of research design B nested data, and explain how these features can be accommodated in multilevel analysis.

### Research design B

**Fig. 2:**
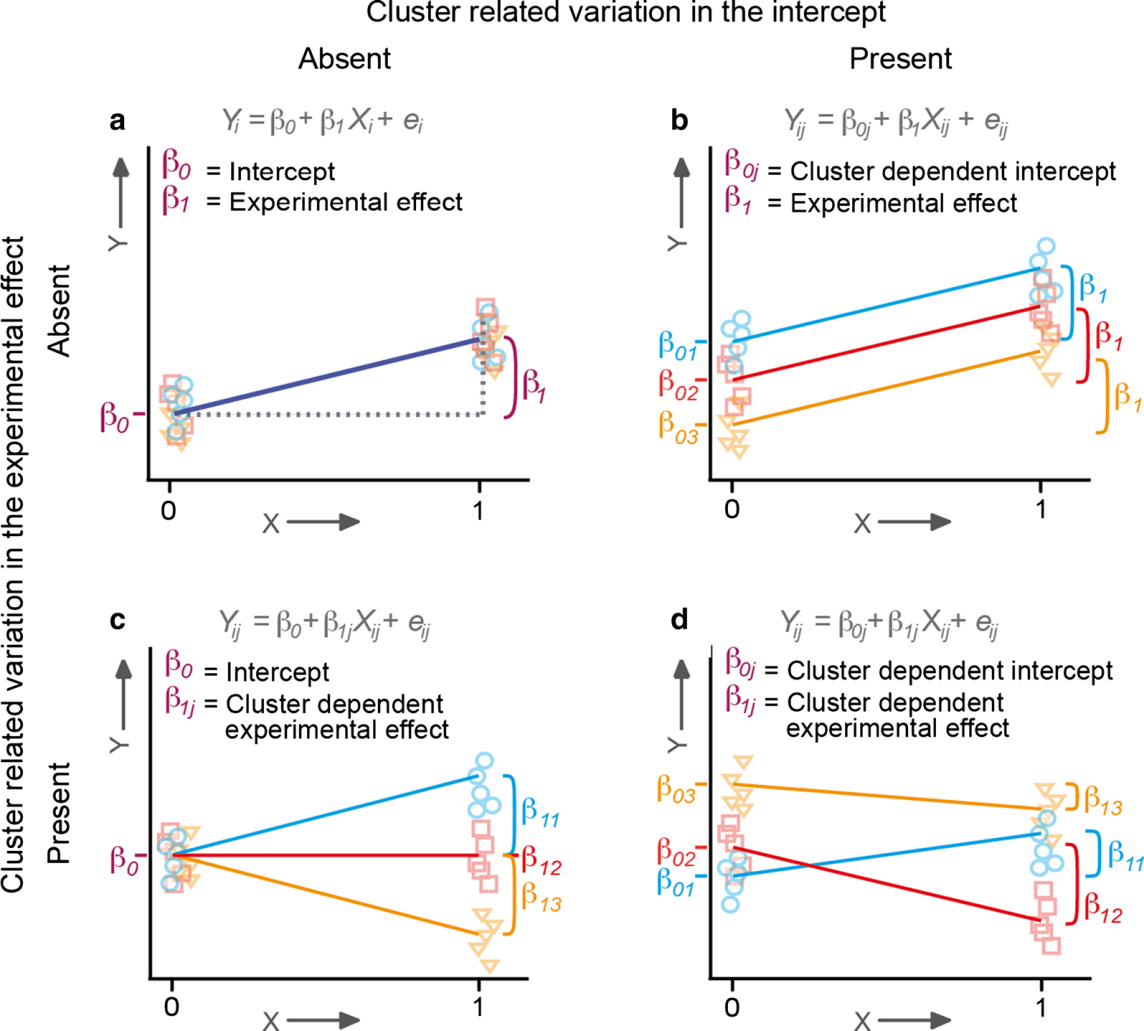
Graphical representations of variants of research design B data. Different possible combinations of cluster-related variation in the mean value of the control condition (i.e., the intercept; $\beta_{0j}$) and cluster-related variation in the experimental effect ($\beta_{1j}$), illustrated for 3 clusters of data: no cluster-related variation (**a**), only cluster-related variation in the intercept (**b**), only cluster-related variation in the experimental effect (**c**), or cluster-related variation in both the intercept and the experimental effect (**d**)

Design B data differ from design A data in that observations collected within the same cluster are allocated to different experimental settings (Fig. 1b). Hence, both the mean value of the dependent variable and the effect of the experimental manipulation may vary over clusters (Fig. 2).

Again one can handle the dependency by calculating a summary statistic per experimental condition per cluster, and then using a standard statistical model (e.g., a paired *t* test or repeated measures ANOVA) to analyze the summary data. For example, when investigating the effect of a growth factor on neurite outgrowth, one could obtain the mean neurite outgrowth for the treated and untreated cells per mouse, and use these in the statistical analysis. However, using such summary statistics is not recommended. Summarizing implies a loss of information, and therefore may result in a loss of statistical power to detect the experimental effect of interest. In addition, this may result in incorrect parameter estimates if the cluster sample sizes vary (even if the summary statistics are weighted by the sample size of the cluster) [8]. Another option in analyzing design B data is to take the cluster effect into account by including it as a factor in standard regression analysis (i.e., fixed effects regression). That is, if there are N clusters, the regression analysis would contain an indicator variable that denotes the experimental condition of the observation, plus *N* − 1 indicator variables that denote cluster membership (in research design A this is not possible: observations from the same cluster all pertain to the same experimental condition, as such there is not enough information to estimate both the cluster effect and the experimental effect). This solution is only practical if the number of clusters is small. Besides, it restricts the statistical inference to the clusters in the study and no other clusters, rendering this approach unsuited for generalization to the population [9]. Specifically, in this approach, the clusters are regarded as fixed, and not as a random sample from the general population, which limits the generalizability of the obtained research results. In addition, this approach does not easily allow quantification of the amount of cluster-related variation in the experimental effect, which can be informative on itself. In multilevel analysis of design B data, the clusters are regarded as random samples from the general population. The comparison between experimental conditions is conducted on the experimental condition specific means within clusters, while including information on the variance within clusters (e.g., differences between observations within a mouse that remain after taking the effect of the experimental effect into consideration) and the variance in the experimental effect over clusters (e.g., differences between mice in the experimental effect). See “Box 2” for a description of the multilevel model for design B. Multilevel analysis uses all available information, can be used with varying cluster sample sizes, allows generalization to the general population, and quantifies the amount of cluster-related variation and is therefore the preferred statistical approach. In multilevel analysis, cluster-related variation in the experimental effect is quantified by the variance of the experimental effect over clusters, which we denote by $\sigma_{u1}^{2}$ (see “Box 2”).

## Box 2: Multilevel model for research design B

To accommodate possible variation in the effect of the experimental manipulation across clusters, the model at the level of the individual observations given in Eq. 1 is extended as follows:

5 $$Y_{ij} = \beta_{0j} + \beta_{1j} *X_{ij} + e_{ij} \quad {\text{with }} \ e_{ij} \sim N(0,\sigma_{e}^{2} ),$$

i.e., the experimental effect, denoted by $\beta_{1j}$, and the variable X_ij_ indicating the experimental condition of observation *Y*_ij_, are now defined at the individual observational level instead of on the cluster level (hence the use of *X* instead of *Z*). The experimental effect $\beta_{1j}$ now accounts for systematic differences between observations *within* a cluster, rather than systematic differences between observations in different clusters. How much observations within a cluster differ between experimental conditions can vary over clusters (denoted by the subscript *j* in $\beta_{1j}$), resulting in a cluster level model for both the intercept $\beta_{0j}$, and the cluster-dependent experimental effect $\beta_{1j}$:

6 $$\beta_{0j} = \gamma_{00} + u_{0j} ,\quad {\text{ and}}$$

7 $$\beta_{1j} = \gamma_{10} + u_{1j} , \quad {\text{ with }} \ \left( {\begin{array}{*{20}c} {u_{0j} } \\ {u_{1j} } \\ \end{array} } \right) \sim N\left( {\begin{array}{*{20}c} {\sigma_{u0}^{2} } & {\sigma_{u0,u1}^{2} } \\ {\sigma_{u0,u1}^{2} } & {\sigma_{u1}^{2} } \\ \end{array} } \right)$$

i.e., the experimental effect $\beta_{1j}$, just like the intercept $\beta_{0j}$, is composed of an overall experimental effect across all clusters $\gamma_{10}$, and a cluster specific deviation from that overall experimental effect, $u_{1j}$. The variance of the experimental effect over clusters is noted by $\sigma_{u1}^{2}$ (i.e., the variance of $u_{1j}$). In this extended model, the parameter $\beta_{0j}$ represents the cluster-specific intercept, which is now interpreted as the mean of the observations belonging to the control condition of that particular cluster (i.e., given that the indicator variable *X*_*ij*_ equals 0 for the control condition). $\beta_{1j}$, in turn, represents the cluster-specific deviation from $\beta_{0j}$ of those observations in the cluster belonging to the experimental condition. The variance of the cluster-dependent experimental effect $\sigma_{u1}^{2}$ is often referred to as the slope variance, as $\beta_{1j}$ is often referred to as the cluster-specific slope parameter.

Note that models that include both intercept variance and slope variance may include a covariance between the random intercept and random slope noted by $\sigma_{u0,u1}^{2}$ (where the term “random” simply implies that the intercept and slope vary across clusters). When comparing a control and an experimental condition using a 0/1 dummy coded indicator, the intercept represents the mean value of the dependent variable *Y* of the control condition, and the slope represents the deviation from this mean value for the experimental condition. In this case a positive covariance between intercept and slope implies that higher values in the control condition coincide with larger experimental effects, while a negative covariance implies that higher values in the control condition coincide with smaller experimental effects. An example of negative covariance is when cells whose neurons show a relatively large value for neurite outgrowth in the control condition, tend to show a smaller effect of the growth factor.

In Additional file 1, a worked example of multilevel analysis of research design B data is presented. A detailed and accessible explanation of multilevel modeling, including details of the statistical analysis, can be found in Hox, Goldstein, and Snijders and Bosker [10–12]. Note that when performing multilevel analysis, a sufficient number of clusters, and observations per cluster, are required to obtain stable and unbiased estimates [13, 14]. To obtain unbiased estimates of the overall experimental effect and its standard error, a minimum of 10 clusters and 5 observations per experimental condition per cluster is recommended. If one also requires accurate estimates of the cluster-related variation in the intercept (i.e., the mean value of the control condition, given that the indicator variable that denotes the experimental condition equals 0 for the control condition, see “Box 2”) and, for research design B specifically, the experimental effect, a minimum of 30 clusters is recommended. Hence, careful planning of the research design is required when dealing with nested data, as multilevel analysis requires sufficient observations on both levels. When the number of clusters is small (e.g., <10), but the number of observations within each cluster is large (e.g., >300), Bayesian estimation methods are an alternative, as these have proven to yield less biased estimates than maximum likelihood approaches in this specific instance [15]. Another option would be to use fixed effects regression, in which the obtained research results are only valid for the clusters in the study.

In the few methodological papers in the neuroscience literature that discussed the analysis of research design B data, the focus has been on the special case that the experimental effect is invariant over clusters [2, 4]. This is a strong assumption, which may not hold. Hence, the possibility that the experimental effect varies over clusters should be taken into account in the statistical model. Intrinsic biological variation, and small differences in the experimental conditions or measurement procedures all may cause differences between clusters, and consequently differences in the experimental effect over clusters. For instance, in the growth factor experiment, not all experiments may be performed using the same batch of growth factor, which can result in variation in the experimental effect.

The failure to accommodate variation in the intercept (i.e., mean value of the control condition), and/or experimental effect over clusters (i.e., dependency) in research design B data can result in incorrect inferences concerning the experimental effect. The exact consequence of ignoring the dependency depends on the effect of clustering. First, if in research design B data clustering is a source of variation in the intercept, but not a source of variation in the experimental effect (Fig. 2b), the failure to accommodate clustering (e.g., by applying a standard statistical model like regression or *t* test), may result in a loss of statistical power to detect the experimental effect^3^ [4, 8]. A correctly specified multilevel model does not incur this loss of power because it effectively accommodates the otherwise unexplained variation in the intercept over clusters, thus providing a better signal-to-noise ratio. Below, we examine the loss in statistical power that may arise when conventional methods are used to analyze research design B data given an experimental effect that does not vary over clusters. We express this loss in power as a function of various characteristics of the data.

Second, if clustering is a source of variation in the experimental effect (Fig. 2c, d), standard errors obtained in standard statistical models and multilevel analysis in which random effects are incorrectly specified, are likely to be underestimated [8]. This results in a downward bias in *p* values, and consequently an inflated Type I error rate (i.e., rate of false positives) that exceeds the nominal $\alpha$-level (e.g., $\alpha = 0.05$). However, the degree of inflation produced in these models, and variation in the Type I error rate as a function of variation in the experimental effect over clusters, has not been demonstrated before. Moreover, little is known about the effect on the Type I error rate associated with standard statistical models and misspecified multilevel analysis, given systematic variation in both experimental effect and the intercept (Fig. 2d).

The aim of this paper is to illustrate by means of simulation how misspecification of the statistical model for research design B data affects the false positive rate and statistical power.

## Methods

We use randomly generated (i.e., simulated) datasets to illustrate the effects of cluster-related variation in design B data on results of various statistical tests. We varied the magnitude of the experimental effect, the amount of cluster-related variation in the intercept and in the experimental effect, and the sample size. We determined how these variables influenced the obtained results. We considered a design with two experimental conditions, which we refer to as the control and the experimental condition. The generated datasets were analyzed using the following four statistical methods: a *t* test on the individual observations (i.e., modeling the data as shown in Fig. 2a), a paired *t* test on the experimental condition specific cluster means, a multilevel model on the individual observations that only accommodates cluster-related variation in the intercept (i.e., modeling the data as shown in Fig. 2b), and a multilevel model on the individual observations that accommodates cluster-related variation in both the intercept and the experimental effect (i.e., modeling the data as shown in Fig. 2d). Note that the standard statistical methods on summary statistics produces correct parameter estimates only if the (sub)sample sizes are equal over clusters and experimental conditions [8]. An overview of the parameter settings for each simulation study is provided in Table 2.

**Table 2:** Parameter settings used to generate the simulated datasets

	Variation in intercept (ICC**)**	*Study*	*Aim of study*	$\sigma_{u1}^{2}$	*ICC*	*d*	*N*	*nc*
*Variation in experimental effect* (${\sigma}_{{{u}1}}^{2}$)								
*Absent*	Absent	1a	Statistical power	0.00	0.00	0.20	10	5–50
						0.50	30	
	Present	1b	Statistical power	0.00	0.25	0.20	10	5–50
					0.50	0.50	30	
*Present*	Absent	2a	False positive rate	0.025	0.00	0.00	50	5–105
				0.050				
				0.150				
	Present	2b	False positive rate	0.025	0.50	0.00	50	5–105
				0.050				
				0.150				

The simulations with no cluster-related variation in the experimental effect (studies 1a and 1b; $\sigma_{u1}^{2}$ = 0) investigate the effect on the statistical power to detect the experimental effect in case that variation in the intercept is present but not accommodated. Hence, in studies 1a and 1b, the magnitude of the overall effect of the experimental manipulation, expressed by effect size *d,* exceeds zero (*d* > 0). The simulations including cluster-related variation in the experimental effect (studies 2a and 2b; $\sigma_{u1}^{2}$ > 0) investigate the effect on the false positive rate in case that this variation in the experimental effect is not accommodated in the statistical model. Hence, in studies 2a and 2b, the magnitude of the overall effect of the experimental manipulation equals zero (*d* = 0)

*ICC* intracluster correlation, denoting the extent of dependency in the data, *N* number of clusters, *nc* number of observations per experimental condition per cluster

First, we illustrate the effect on the statistical power to detect the overall experimental effect in the specific case that variation in the intercept is absent, or present but not accommodated, and cluster-related variation in the experimental effect is absent (i.e., study 1a and 1b in Table 2). That is, we ask: if data is generated according to Fig. 2a, b, how does the statistical power compare across the four statistical methods (i.e., the *t* test on the individual observations, the paired *t* test on summary statistics, and the two types of multilevel analysis)?

Second, we illustrate the effects of the presence of cluster-related variation in the experimental effect, in combination with either absent or present cluster-related variation in the intercept, on the false positive rate of the experimental effect (i.e., study 2a and 2b in Table 2). That is, we ask: if data is generated such that overall, i.e., taken over all clusters, the experimental manipulation has no effect, but the data includes cluster-related variation in the experimental effect, what is effect on the false positive rate? We illustrate this in case that the data includes no cluster-related variation in the intercept (Fig. 2c), or does include cluster-related variation in the intercept (Fig. 2d), and compare the false positive rate across the four statistical methods (i.e., the *t* test on the individual observations, the paired *t* test on summary statistics, and the two types of multilevel analysis).

For all scenarios, we generated 10,000 datasets. To establish statistical power in studies 1a and b, and the empirical false positive rate in studies 2a and 2b, we counted the number of times that the overall experimental effect was found to be statistically significant given $\alpha$ = 0.05. The datasets were generated such that the experimental effect is expressed in terms of the effect size *d* (obtained by difference between experimental and control condition/within cluster standard deviation $\sigma_{e}$ [16]), where we considered the effects 0.20, 0.50, and 0.80 to be small, medium, and large, respectively [17]. Condition was dummy coded 0 (control) and 1 (experimental), such that the amount of cluster-related variation in the experimental effect $\sigma_{u1}^{2}$ could be interpreted according to the guidelines of Raudenbush and Liu [7]. Accordingly, $\sigma_{u1}^{2}$ values of 0.05, 0.10, and 0.15 are considered small, medium, and large, respectively.

To understand the amount of variation in the experimental effect, consider a medium experimental effect of *d* = 0.50. If the variation in the experimental effect is small, i.e., $\sigma_{u1}^{2}$ = 0.05, this corresponds to a standard deviation of ~0.22. Assuming normally distributed cluster specific deviations from the overall effect size, $\beta_{1j}$, ~95 % of the cluster-specific experimental effects would lie between ~0.07 and ~0.93 (i.e., 0.50 − 1.96 × 0.22 and 0.50 + 1.96 × 0.22, respectively). Using the dummy coding 0 and 1 also ensures that the intercept variance equals the cluster-related variation in the intercept of the control condition in case that both the intercept and the experimental effect show cluster-related variation. The covariance between the intercept and the experimental effect was set to zero in all simulations.

All simulations were performed in R 2.15.3 [18], and multilevel models where fitted using the R package lme4 [19]. The R-code is available upon request from the corresponding author.

## Results and discussion

### Ignoring cluster-related variation can result in interpretational errors

Our simulation results showed that the failure to accommodate the cluster-related variation in either intercept or slope (i.e., in either the mean value of the control condition or the experimental effect) can result in interpretational errors. A general overview of all results is given in Table 3. Below, we discuss the results of studies 1a and 1b (i.e., no variation in the experimental effect) and studies 2a and 2b (i.e., variation in the experimental effect) in detail.

**Table 3:** Consequences of not accommodating cluster-related variation in research design B

	Statistical test	Variation in intercept	
		Absent	Present
		*Statistical power* ^a^	
		*Study 1a*	*Study 1b*
*Variation in experimental effect*			
* Absent*	*T* test ind. obs. *T* test summary st. Multilevel analysis I Multilevel analysis II	Correct Decreased power Correct Correct	Decreased power Decreased power Correct Correct
		*False positive rate*	
		*Study 2a*	*Study 2b*
* Present*	*T* test ind. obs *T* test summary st. Multilevel analysis I Multilevel analysis II	Increased false positive rate Correct Increased false positive rate Correct	Increased false positive rate Correct Increased false positive rate Correct

The results of four statistical tests to detect the experimental effect are compared with respect to (1) statistical power to detect the (overall) experimental effect (when variation in the experimental effect is absent) and (2) false positive rate (when variation in the experimental effect is present). Fitted statistical models are a *t* test on individual observations (*T* test ind. obs), a paired *t* test on the experimental condition specific cluster means (*T* test summary st.), a multilevel analysis that does not accommodate the variation in the experimental effect but does accommodate variation in the intercept (Multilevel analysis I), and a multilevel analysis that accommodates both variation in the intercept and in the experimental effect (Multilevel analysis II)

^a^In case that variation in the experimental effect is absent, all fitted statistical models result in a false positive rate that does not exceed the nominal *α* specified by the user (i.e., correct or slightly conservative, see e.g. [4, 8])

### Ignoring variation in the intercept in design B data can decrease statistical power

The obtained results are equal for the multilevel model that only includes variation in the intercept, and the multilevel model that includes variation in both the intercept and the experimental effect. Therefore, we do not differentiate between the two types of multilevel analysis in this section.

**Fig. 3:**
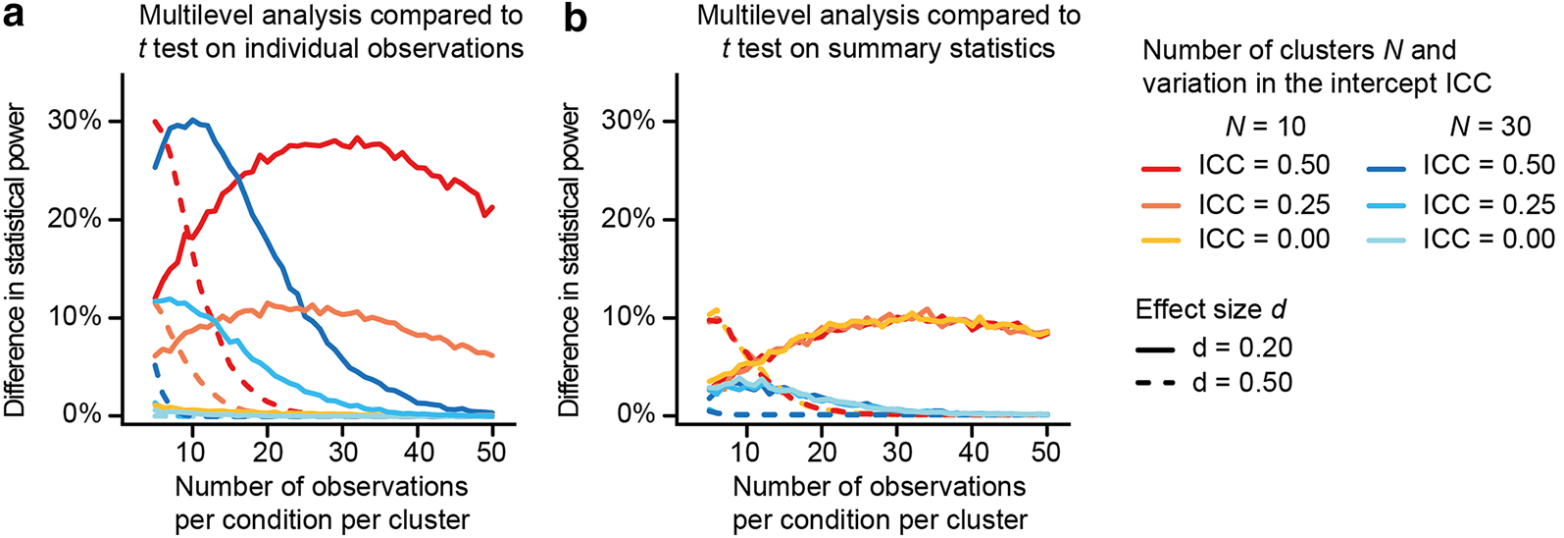
Use of conventional analysis methods on design B data can result in a loss of power. Using conventional analysis methods to model design B data that includes cluster-related variation in the intercept and no cluster-related variation in the experimental effect ($\sigma_{u0}^{2}$ >0 and $\sigma_{u1}^{2}$ = 0; study 1b) results in a loss of statistical power compared to using a multilevel model. The presented results are equal for the multilevel model that only includes variation in the intercept, and the multilevel model that includes variation in both the intercept and the experimental effect. Fitted conventional analysis methods were **a** a *t *test on individual observations and **b** a paired *t* test on the experimental condition specific cluster means. The loss in statistical power is overall greatest when both the number of clusters and effect size *d* are small and the cluster-related variation in the intercept is considerable. In case that the cluster-related variation in the intercept and in the experimental effect both equal zero (that is, ICC = $\sigma_{u1}^{2}$ = 0; study 1a), using a *t* test on individual observations is equally powerful as multilevel analysis, but using multilevel analysis is more powerful compared to a paired *t* test on summary statistics. The actual statistical power of multilevel analysis given $\sigma_{u1}^{2}$ = 0, = 0.20 or 0.50, *N* = 10, and increasing numbers of observations per experimental effect per cluster is given in Fig. 5b, *solid line*

In case of design B data that includes no cluster-related variation in the intercept or experimental effect (i.e., study 1a), conventional statistical analysis (i.e., a *t* test) on individual observations is equally powerful as multilevel analysis, but using multilevel analysis is more powerful compared to conventional statistical analysis (i.e., a paired *t* test) on summary statistics (Fig. 3). The loss in statistical power when using conventional statistical analysis on summary statistics is only present when the number of clusters is small (i.e., *N* = 10).

Analyzing design B data that only includes cluster-related variation in the intercept (i.e., study 1b) using conventional statistical analysis (e.g., a *t *test on individual observations or a paired t test on experimental condition specific cluster means) sometimes results in a loss of statistical power. Compared to using a *t* test on individual observations, the difference in statistical power is greatest when both the number of clusters and the magnitude of the experimental effect are small, and the amount of cluster—related variation in the intercept is large (Fig. 3). For example, in case of substantial cluster-related variation in the intercept giving rise to ICC = 0.50, using a *t* test on individual observations is ~25 % less powerful than multilevel analysis, given 10 clusters and an effect size of 0.20. In case that the overall experimental effect is medium (i.e., *d* = 0.50), multilevel analysis only results in more statistical power given substantial cluster-related variation in the intercept (i.e., ICC = 0.50), and a small number of clusters and small number of observations per experimental condition. Compared to using a paired *t* test on experimental condition specific cluster means, the loss in statistical power compared to multilevel analysis is only present when the number of clusters is small (i.e., *N* = 10), and does not depend on the amount of cluster-related variation in the intercept.

The occasionally observed increase in loss of power as function of increasing number of observations per experimental condition per cluster is due to the fact that multilevel analysis gains in power with increasing number of observations per experimental condition per cluster. The observed decrease in loss of power when the number of observations per experimental condition per cluster increases, is due to the fact that multilevel analysis approximates the maximum power of 100 %, and thus the difference in statistical power between multilevel analysis and conventional analysis methods becomes smaller. The actual statistical power of multilevel analysis given no cluster-related variation in the experimental effect, an effect size *d* of 0.20 or 0.50, 10 clusters, and increasing numbers of observations per experimental effect per cluster is provided in Fig. 5b (solid line; note that the ICC does not influence the power of multilevel analysis to detect the overall experimental effect in case of design B data, and as such does not feature in this figure).

In summary, the failure to take into account the hierarchical nature of data or using summary statistics, results in a loss of power to detect the experimental effect, especially when both the number of clusters and the overall effect are small. Neuroscience studies often report small effects, and may be underpowered due to small sample size [20]. Multilevel analysis of research design B data can increase statistical power compared to conventional analyses, unless of course the statistical power of the conventional analysis approaches 1.

### Ignoring variation in the experimental effect increases the false positive rate

**Fig. 4:**
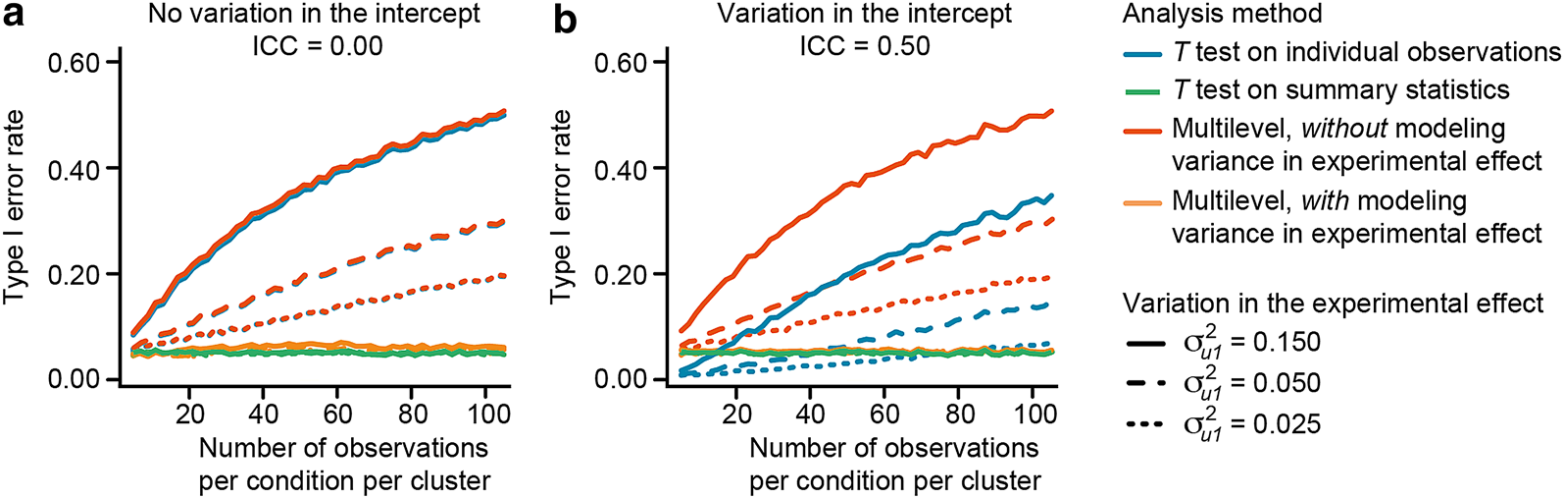
Ignoring variation in the experimental effect results in inflated false positive (i.e., Type I error) rate. Inflation of the Type I error rate already occurs when a small amount of variation in the experimental effect (e.g., $\sigma_{u1}^{2}$ = 0.025) remains unaccounted for in the statistical model, and occurs both when the intercept (i.e., mean value of the control condition) is invariant over clusters (**a**; ICC = 0; study 2a**)**, and when the intercept varies substantially over clusters (**b**; ICC = 0.50; study 2b). In* panel* **a**, the* lines *depicting conventional analysis (i.e., *t* test on individual observations) and misspecified multilevel analysis completely overlap. Using a paired *t *test on the experimental condition specific cluster means results in a correct Type I error rate. In* panel* **b**, the* lines *depicting the paired *t* test and the correctly specified multilevel analysis completely overlap

Given clustering with respect to the experimental effect, the use of a statistical model on individual observations that does not accommodate this variation results in an inflated false positive (i.e., Type I error) rate. First, when variation in the intercept is absent (i.e., study 2a), ignoring variation in the experimental effect results in an actual false positive rate as high as ~20–~50 % (Fig. 4a), depending on the number of observations per cluster and the amount of variation in the experimental effect. Specifically, if the overall experimental effect is zero, both a conventional *t* test and misspecified multilevel analysis (i.e., one that ignores variation in the experimental effect but does model variation in the intercept), yield similarly inflated Type I error rates (lines fully overlap in Fig. 4a). Even a very small amount of cluster-related variation in the experimental effect (i.e., $\sigma_{u1}^{2}$ = 0.025) can results in a Type I error rate of ~20 % if it is not accommodated in the statistical model. In summary, the failure to accommodate cluster-related variation in the experimental effect results in a substantial inflation of the Type I error rate, and this inflation is considerable even when variation in the experimental effect is small.

Second, when variation is present in both the intercept and the experimental effect (i.e., study 2b), accommodating only the cluster-related variation in the intercept (i.e., a misspecified multilevel analysis), or not accommodating cluster-related variation at all (i.e., conventional *t* test) again results in an inflated Type I error rate (Fig. 4b). If the variation in the intercept is large (ICC = 0.50), the Type I error rate increases up to approximately 35 % when using a conventional *t* test. When using a multilevel analysis that only accommodates cluster-related variation in the intercept, the inflation in the Type I error increases up to approximately 50 %. In summary, the substantial inflation of the Type I error rate that arises if cluster-related variation in the experimental effect is not accommodated arises irrespective of the presence of variation in the intercept.

Accommodating cluster-related variation in the experimental effect by either using correctly specified multilevel analysis or using conventional models on summary statistics (i.e., a paired *t* test on the experimental condition specific cluster means), does result in a correct Type I error rate (i.e., study 2a and 2b). See “Box 3” for a detailed explanation of why ignoring cluster-related variation in the experimental effect results in an increased false positive rate.

## Box 3: Inflation of the false positive rate in research design B

By considering the standard error of the overall experimental effect in multilevel analysis, $SE_{{\gamma_{10} }}$, we can clarify why increasing cluster-related variation in the experimental effect $\sigma_{u1}^{2}$ and/or increasing sample size per cluster *nc* (given that $\sigma_{u1}^{2}$ > 0) results in an inflated Type I error in conventional analysis (i.e., *t* test) on individual observations in nested data. In multilevel analysis, (adjusted from Eq. 13 in [7])

8 $$SE_{{\gamma_{10} }} = \sqrt {\frac{{nc*\sigma_{u1}^{2} + \sigma_{e}^{2} }}{nc*N}}$$

where *N* denotes the number of clusters, and $\sigma_{e}^{2}$ denotes the residual error variance. In the *t* test, in contrast, the standard error of the experimental effect, denoted as $SE_{{\beta_{1} }}$, is

9 $$SE_{{\beta_{1} }} = \sqrt {\frac{{\sigma_{e}^{2} }}{nc*N}} .$$

Comparing Eqs. 8 and 9, we see that ignoring non-zero cluster-related variation in the experimental effect, $\sigma_{u1}^{2}$, results in an underestimation of $SE_{{\beta_{1} }}$, and consequently in downward biased p values. The degree of underestimation depends on the number of observations per cluster *nc*, and on the amount of cluster-related variation in the experimental effect, $\sigma_{u1}^{2}$.Important to note is that, first, Eqs. 8 and 9 hold in the case of standardized data [i.e., both the experimental variable *X* and the outcome variable *Y* are standardized such that ~*N*(0,1)] and a balanced design (i.e., the number of observations per condition per cluster is equal over clusters). Secondly, in Eq. 9, $\sigma_{e}^{2}$ is actually a composite of all sources of variation that remain unexplained in the conventional analysis model, i.e., the actual residual error variance, but also the intercept variance and the variance in the experimental effect over clusters. Thirdly, the intercept variance $\sigma_{u0}^{2}$ plays no role in Eq. 8. This explains why the Type I error rate of the misspecified multilevel model in simulation studies 2a and 2b (i.e., simulated data that includes only cluster-related variation in the experimental effect, and simulated data that includes both cluster-related variation in the intercept and experimental effect, respectively, see Table 2 for exact parameter settings) is unaffected by the value of the ICC. That is, the obtained Type I error rate of the misspecified multilevel model is equal for the simulation studies that do and do not include cluster-related variation in the intercept (see Fig. 4a, b).

### Explaining part of the variation in the experimental effect: increasing both theoretical insights and power

The simulation studies have shown that ignoring cluster-related variation in the intercept ($\sigma_{u0}^{2}$) and experimental effect ($\sigma_{u1}^{2}$) may result in incorrect inference concerning the experimental effect. However, it is important to emphasize that these variance terms are not merely “noise” i.e., nuisance parameters: they can advance our biological understanding, and can be of practical interest. Here, we focus specifically on the information that can be obtained from cluster-related variation in the experimental effect.

Variation in the experimental effect is informative about the generalizability of the experimental effect [7]: is the impact of the experimental manipulation similar across (biologically intrinsic) different settings? In some instances, sources of cluster variation may be difficult to measure. For example, when investigating a feature at the cellular level in neurons taken from mouse embryos, it is conceivable that not all neurons were harvested at the exact same embryonic age, resulting in different levels of neuron maturation. The variation in neuronal maturation may in turn influence the magnitude of the effect of the experimental manipulation. In this case, multilevel modeling accommodates the variation in outcome due to different levels of neuron maturation, despite the fact that neuron maturation is not explicitly measured. An added advantage arises when possible sources of cluster-related variation can be recorded: they can be used to (partly) explain the cluster-related differences in the experimental effect by including them in the model (see below). As such, recorded sources of cluster variation can facilitate the understanding of the conditions in which the experimental manipulation does or does not have an effect, and thus of the generalizability of this effect. For instance, suppose pregnant mice are administered fluoxetine in their food, which induces life-long cortical abnormalities in the pups. The food intake, and therefore drug intake, which may vary between mice, can easily be measured. If we are interested in a certain drug B that is hypothesized to counteract these developmental changes upon treatment of the pups, we can administer drug B to half of the pups in each nest, and use the remaining pups as controls. Besides the counteractive effect of drug B on drug A, we may investigate whether a measure of food (i.e., fluoxetine) intake in the mothers explains some of the variation observed in the effect of drug B on the (severity of the) cortical abnormalities. In this case, we might learn that the extent to which drug B can alleviate the detrimental effects of fluoxetine depends on the level of fluoxetine exposure.

Explaining variation in the experimental effect by one or more variables is achieved by adding those variables as covariates to the model. The broad definition of a covariate is a variable that is used to adjust the predicted outcome *Y* for differences associated with the covariate, which is measured before (or simultaneous with) the outcome variable *Y*, and correlates with *Y* [21]. Note that in order to (partly) explain the cluster-related differences in the experimental effect, one needs a *cluster*-*level* covariate, like food intake of the mother mouse in the current example.

Besides the fact that explaining part of the variation in the experimental effect can advance our biological understanding, adding a relevant covariate to the statistical model can also increase the statistical power to detect the overall experimental effect. Specifically, by (partly) accounting for the cluster-related variation in the experimental effect, the remaining unexplained cluster-related variation in the experimental effect $\sigma_{u1}^{2}$ decreases. As shown in Eq. 8 in “Box 3”, a decreased $\sigma_{u1}^{2}$ results in a decreased standard error of the overall experimental effect, and hence an increased statistical power to detect this effect. As such, adding a cluster-level covariate to the model can be of practical interest, as it can boost statistical power without, or additional to, increasing sample size.

There are, however, some considerations regarding the inclusion of covariates. First, in case that the statistical model includes covariates, the estimated value, and hence the interpretation, of the experimental effect is conditional on the covariates. In our example, the research question would change from “Does drug B counteract the cortical abnormalities caused by prenatal fluoxetine exposure?” to “When correcting for relative differences in prenatal fluoxetine exposure, does drug B counteract cortical abnormalities caused by prenatal fluoxetine exposure?”. Note that when the covariate is a design variable, e.g., batch number, conditional interpretation of the experimental effect is biologically no different from the unconditional interpretation: we simply correct for measurement noise that we are not interested in.

**Fig. 5:**
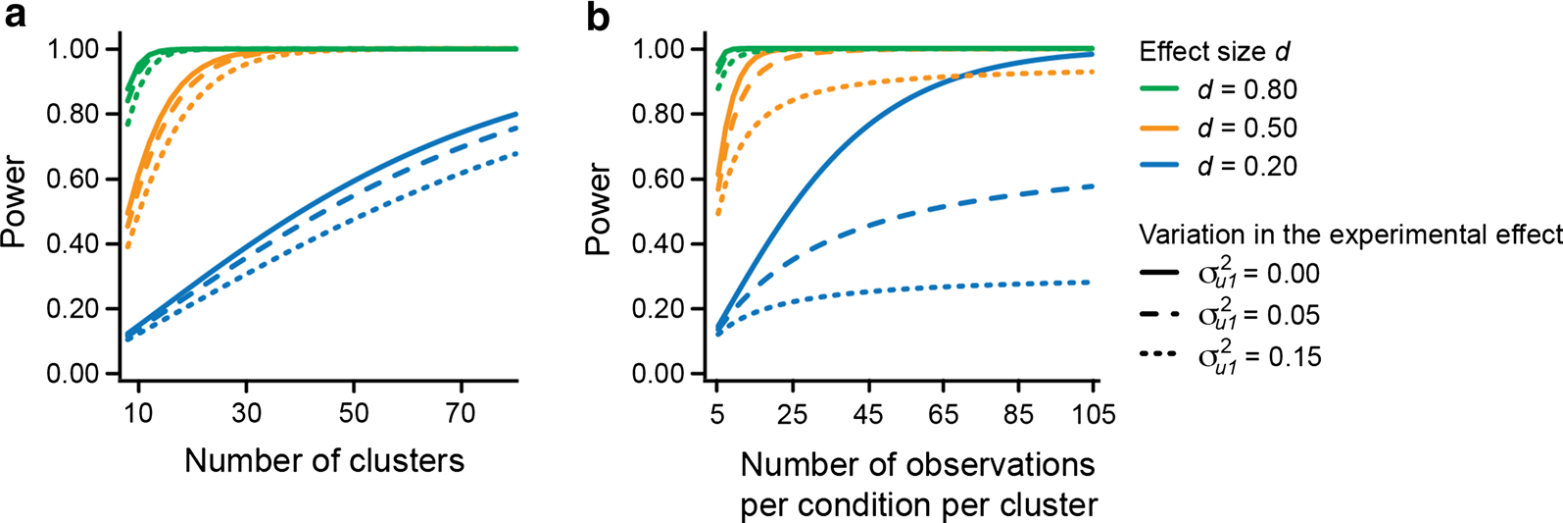
Power of multilevel analysis to detect the overall experimental effect in research design B. Power is depicted in nine conditions (effect size *d* of 0.20, 0.50, or 0.80, and cluster-related variation in the experimental effect of 0.00, 0.05, and 0.15) and as function of the number of clusters (**a**) or the number of observations per cluster per condition (**b**). In both **a** and **b**, two experimental conditions are compared, using a balanced research design. As the cluster-related variation in the intercept in research design B does not influence the statistical power to detect the overall experimental effect (see Eq. 8 in “Box 3”), the ICC does not feature in this *figure*. In **a**, the number of observations is held constant at 5 observations per condition in each cluster; in **b**, the number of clusters is held constant at 10. Evidently, the number of clusters, and not the number of observations per cluster, is essential to increase the statistical power to detect the experimental effect

Second, if a relevant covariate is not measured routinely within the experimental setup, but needs to be measured specifically, this may involve an increase in research costs. If the sole reason to include the covariate is to increase statistical power (and not to facilitate biological understanding), one needs to consider the return in power of these costs [10]. Also, how much power is gained by decreasing the unexplained variation in the experimental effect depends on the allocation of sample sizes over clusters, and on number of observations per cluster (for example, when comparing $\sigma_{u1}^{2}$ = 0.05 and 0.15 in panel b of Fig. 5, one can see that the difference in power for $\sigma_{u1}^{2}$ = 0.05 and 0.15 is smaller when the number of observations per cluster is smaller, and larger when the number of observations per cluster is larger). The program PinT [22] can be used to evaluate how much a particular covariate increases power given the amount of explained variation in the experimental effect and the allocation of sample sizes. In addition, one has to keep in mind that sample size can put a limit on how many parameters, hence covariates, can be added to the statistical model. So careful planning of the study, including the intended covariates, is advised.

Third, one can also attempt to increase power by including a covariate at the level of the individual observations. A covariate at the level of the individual observations can increase statistical power if it (partly) explains why observations within a condition within a cluster vary with respect to the dependent value. This decreases the residual error variance $\sigma_{e}^{2}$ of the model, and hence increases the statistical power to detect the overall experimental effect. For example, when performing siRNA-mediated knockdown in neurons, one could measure knockdown efficiency for each neuron besides the neuronal measurement of interest. One can observe in Eq. 8 in “Box 3”, however, that a reduced (unexplained) variation in the experimental effect $\sigma_{u1}^{2}$ has a greater effect on decreasing the estimated standard error of the overall experimental effect than a reduced residual error variance $\sigma_{e}^{2}$. Therefore, including a covariate at the individual observation level to increase statistical power is only advisable if the covariate is expected to explain a considerable amount of variation within condition within clusters.

### Maximizing power by optimally allocating sample sizes

In conventional analyses, ensuring sufficient statistical power to detect the experimental effect of interest is usually accomplished by calculating the required total number of observations to collect. When it comes to statistical power in multilevel analyses, however, one has to determine the sample size at two levels: the sample size at the individual level, i.e., the number of observations per cluster, and the sample size at the cluster level, i.e., the number of clusters. The number of observations per cluster, and the number of clusters, do not affect statistical power equally, and are often not equal in costs. Hence, a key question is how to optimally allocate observations over clusters, balancing both power and costs. An excellent account of power in multilevel analysis in case of design B and a cost-benefit analysis of power is provided in [7]. For a balanced (i.e., the number of observations per condition are equal both between conditions and between clusters) 2-level multilevel model without covariates, we provide a brief summary in Additional file 2, which includes an explanation of how to calculate the estimated power for a given number of observations per cluster *n*, number of clusters *N*, and choices of other key parameters in the model.

In design B, a larger number of observations per cluster provides precision on the estimate of the experimental effect within a cluster, while a larger number of clusters provides precision on the overall experimental effect. Hence, the power to detect the overall experimental effect benefits most from increasing the number of clusters *N*. This is illustrated in Fig. 5: the statistical power to detect the overall experimental effect steadily increases to 100 % as the number of clusters increases (Fig. 5a), while increasing the number of observations per cluster per condition sometimes results in a plateau (much) lower than 100 % (Fig. 5b). How much power increases as a result of extra observations per clusters depends on the amount of cluster-related variation in the experimental effect.

## Conclusions

To draw valid conclusions in a nested experimental design, it is crucial to use the appropriate statistical method. We showed previously that design A data (i.e., nested data that possibly show cluster-related variation in the intercept) are abundant in neuroscience literature, and that proper statistical analysis of such data is crucial to avoid false positives [1].

Here, we showed that in case of design B data (i.e., nested data that possibly show cluster-related variation both in the intercept and in the experimental effect), correct statistical modeling of such data is also critical to avoid incorrect inference. However, in case of design B data, the exact consequences of ignoring the dependency depend on the nature of clustering. If cluster-related variation in the experimental effect is present, not accommodating this cluster-related variation results in an inflated false positive rate. That is, in design A data, not accommodated variation in the intercept results in an inflated false positive rate, while in design B data variation in the experimental effect causes inflation in the false positive rate when not accommodated. Importantly, inflation of the false positive rate already occurs with a small amount of cluster-related variation in the experimental effect. In addition, if cluster-related variation is limited to the intercept (and absent in the experimental effect), failure to correctly accommodate this variation can result in a loss of statistical power to detect the experimental effect of interest. The loss in statistical power when using conventional analysis methods (i.e., *t *test) on individual observations instead of correctly specified multilevel analysis is noteworthy when both the number of clusters and the overall effect are small. In addition, we showed that using standard statistical methods on summary statistics (i.e., paired *t *test) does result in a correct false positive rate, but results in a loss of statistical power to detect the experimental effects of interest when the number of clusters is small. Importantly, the use of standard statistical methods on summary statistics only results in correct parameter estimates if the (sub)sample sizes are equal over clusters and experimental conditions (even if the summary statistics are weighted by the sample size of the cluster) [8].

Finally, multilevel analysis can provide valuable insight into the generalizability of the experimental effect over (biologically intrinsic) varying settings, and can be used to utilize cluster-related information to explain part of the variation in the experimental effect. Therefore, multilevel analysis not only ensures correct statistical interpretation of the results, and thus correct conclusions, but can also provide unique information on the collected research data that cannot be obtained when standard statistical methods are used on either individual observations or summary statistics.

## Additional files

10.1186/s12868-015-0228-5 Effect of neurite location (axon/dendrite) on traveling speed of intracellular vesicles: a worked example. An example of multilevel analysis of research design B data, including syntax to perform multilevel analysis in the statistical packages SPSS and R.
10.1186/s12868-015-0228-5 Calculating the optimal allocation of sample sizes and estimating statistical power to detect the overall experimental effect. Explanation on how to calculate the optimal allocation of sample sizes over clusters and within clusters given the available resources, and explanation on how to estimate power for a balanced (i.e., the number of observations per condition are equal both between conditions and between clusters) 2-level multilevel model without covariates.

## Abbreviations

ICC: intracluster correlation
PinT: power in two-level designs

## Footnotes

1 In the context of RCBD, the term “nested” is used to describe how experimental manipulations or treatments are combined. Specifically, treatments are referred to as nested if the various experimental conditions of treatment B do not appear with every level of treatment A (e.g., conditions B1 and B2 are only observed in combination with A1, while B3 and B4 are only observed with A2) [23]. In multilevel literature, however, the term nesting is used to describe the data. Specifically, nested data are characterized by a hierarchical, multi-level structure in which individual observations are clustered, or nested, within hierarchically higher organized groups or clusters [10].

2 Let m_i_ denote the true mean in the cluster *i*, and s denote the true within-cluster standard deviation, assumed to be equal for all clusters (hence no subscript). Let m_i_ and s_i_ denote the estimated mean and standard deviation in cluster *i* based on *n*_i_ observations. The standard error of the mean m_i_ equals s.e.(m_i_) = s_i_/√(*n*_i_). If all m_i_ in a given experimental condition (e.g., the WT mice) are equal across clusters, s.e.(m_i_) reflects the variation in cluster means that is solely due to random sampling fluctuation. In that case, the data can be viewed as independent, and can be analyzed using standard statistical models. However, due to systematic differences between the clusters, clustering often gives rise to variation in m_i_ that exceeds this random sampling fluctuation. In the case that data display such (cluster-related) dependency, multilevel analysis is called for. The systematic differences between clusters may be due to known (possibly measured) or unknown factors.

3 In a previous paper specifically on design A data, we showed that not accommodating variation in the intercept results in an increased false positive rate. To avoid confusion on the effect of not accommodating variation in the cluster-related intercept, let us note the following. In design A data, the experimental effect is at the cluster level and thus explains systematic differences between clusters. In this case, variation in the intercept thus represents variation in the outcome of one of the experimental conditions. When this variation in the outcome is not taken into account, this results in a too precise estimate of the experimental effect (i.e., downward biased standard error), and hence an increased false positive rate. In design B data however, the experimental effect is at the level of the individual observations, and is thus explains systematic differences *within* clusters and hence determined within each cluster separately. Variation in the intercept (i.e., the mean value of the control condition) here represents fluctuations that do not influence the size of the experimental effect within each cluster. Not accommodating variation in the intercept in research design B thus results in a lower signal to noise ratio, hence decreased statistical power, instead of a higher false positive rate.
