# Supplementary material for: Multilevel analysis quantifies variation in the experimental effect while optimizing power and preventing false positives
Source: BMC Neurosci. 2015 Dec 19;16:94. doi: 10.1186/s12868-015-0228-5 (PMC4684932; doi:10.1186/s12868-015-0228-5)
Supplement: Supplementary file 1 — 10.1186/s12868-015-0228-5 Effect of neurite location (axon/dendrite) on traveling speed of intracellular vesicles: a worked example. An example of multilevel analysis of research design B data, including syntax to perform multilevel analysis in the statistical packages SPSS and R. [file 12868_2015_228_MOESM1_ESM.pdf]

Additional file 1

Effect of neurite location (axon/dendrite) on traveling speed of intracellular vesicles: a worked example

To clarify the procedure of a multilevel analysis, we use a hypothetical example in which measurements of velocity of intracellular vesicles are nested within neurons ($Neuron\_ID$). In this example, we investigate whether velocity differs between axonal and dendritic measurements (*Location*). Collected from 20 neurons, there are on average 100 measurements per neuron (44 to 58 axonal measurements [*Location* = 0], and 43 to 56 dendritic measurements [*Location* = 1]), resulting in a total of 2000 measurements on velocity.

The outcome variable velocity is standardized ($ZVelocity$; i.e., the variable is transformed such that it has a mean of 0 and a standard deviation of 1). Standardized variables are easily obtained in e.g. SPSS (`Analyze` → `Descriptive Statistics` → `Descriptives`: select the variables you want to standardize and tick the box "Save standardized values as variables"). *Location* is dummy coded 0 (axonal) and 1 (dendritic). The advantage of using data in which the outcome variable is standardized and the dummy indicator is coded as 0 and 1, is that the amount of neuron-related variation in the experimental effect σ^2_{u1} can be interpreted according to the guidelines of Raudenbush and Liu [1]. Using these conventions, values of σ^2_{u1} equaling 0.05, 0.10, and 0.15 are considered small, medium, and large, respectively. An added advantage of using standardized data is that the intercept variance σ^2_{u0} approximates the ICC, and an added advantage of using the dummy coding 0 and 1 is that the intercept variance σ^2_{u0} equals the cluster-related variation in the mean value of axonal measures (i.e., the condition coded as 0).

We illustrate multilevel analysis using the statistical package SPSS, and syntax is provided for each step. To illustrate how the same analyses can be run in R, corresponding R code is provided at the end of the document.

Assumptions

One of the assumptions of standard multilevel analysis is that the outcome variable is normally distributed. A visual inspection of the distribution of $ZVelocity$ for the axonal and dendritic measurements separately shows that $ZVelocity$ can be considered normally distributed. When data are non-normal, transformations can be considered, or a multilevel model for non-normal data can be used (i.e., SPSS also allows for multilevel analysis of dichotomous and Poisson distributed outcome variables). When the results of multilevel analysis of the transformed and untransformed data are similar, interpreting the results of the untransformed data can be easier, and is therefore recommended. Another assumption concerns the absence of outliers, i.e., standardized values below -3.33 and above 3.33 (assuming standard normally distributed data) need to be excluded from the analysis.

Analysis

Multilevel analysis is conducted in a stepwise manner, building up the model from simple to more complex. Before conducting the actual multilevel analysis, we will first visually examine the variance between and within neurons of the measured velocities to get an idea of the degree of relative similarity between observations

Table S1 Syntax and selected output for visualization of vesicle velocity over neurons

```
GRAPH
/SCATTERPLOT(BIVAR)=Neuron_ID WITH ZVelocity BY Location
/MISSING=LISTWISE.
```

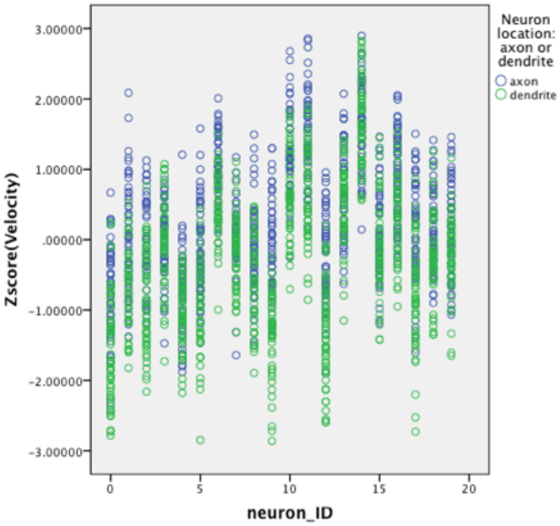

obtained from the same neuron, and how much the difference between axonal and dendritic measurements varies over neurons. We plot the measured velocities for each neuron separately and color code the distinct measurements from the axon and dendrites: syntax and output are shown in Table S1. The figure shows that there is considerable variation in both axonal and dendritic measurements, both within and between neurons. In addition, the difference in velocity between axonal and dendritic measurements varies over neurons: in some neurons the measured velocity of axonal and dendritic vesicles completely overlap, and in others they do not. In general, however, the velocity seems slightly lower for dendritic measurements, but we of course need to test this.

Intercept only model In order to perform the analysis, one additional variable has to be created: an artificial intercept (*int*), which is a variable that always has value 1. Next, an estimate of the intracluster correlation (ICC) can be obtained by running an intercept only model (see equation 1 in Box 1), i.e., a model in which every neuron is allowed to have its own mean velocity, but that does not include Location as experimental variable: syntax and selected output are presented in Table S2.

In the intercept only model, the intercept represents the overall mean value for velocity, i.e., velocity calculated across all cells and across both axonal and dendritic measures. As we standardized the variable velocity, the overall mean is zero. In the Table "Estimates of covariance parameters" we see that the variation in the mean velocity over neurons equals .505 (i.e., the intercept has a variance of .505, suggesting that the mean velocity shows variation between neurons). To obtain an estimate of the intracluster correlation (ICC; a standardized measure of the variation of the

Table S2 Syntax and selected output for intercept-only model through SPSS MIXED

```
COMPUTE int = 1
EXECUTE.

MIXED
ZVelocity with int
/fixed int | noint
/random int | subject(Neuron_ID) covtype(un)
/METHOD = ML
/print solution testcov r.
```

Estimates of Fixed Effects[a]

Parameter	Estimate	Std. Error	df	t	Sig.	95% Confidence Interval	
						Lower Bound	Upper Bound
int	.000000	.159742	20.000	.000	1.000	-.333217	.333217

a. Dependent Variable: Zscore(Velocity).

Covariance Parameters

Estimates of Covariance Parameters[a]

Parameter		Estimate	Std. Error	Wald Z	Sig.	95% Confidence Interval	
						Lower Bound	Upper Bound
Residual		.494089	.015703	31.464	.000	.464250	.525845
int [subject = Neuron_ID]	Variance	.505411	.161388	3.132	.002	.270296	.945040

a. Dependent Variable: Zscore(Velocity).

mean value over neurons), apply equation 3 in the main text:

$$ICC = \frac{\sigma_{u0}^2}{\sigma_{u0}^2 + \sigma_e^2} = \frac{0.505}{0.505 + 0.494} = 0.506. \quad (1)$$

This means that when only considering the mean $ZVelocity$ of each neuron (i.e., not distinguishing between $Location$), 50.6% of the variability in $ZVelocity$ is due to differences between neurons, i.e., can be explained by neuron-membership. Note that because the outcome variable vesicle velocity is standardized in our model, the variance estimate of the intercept (.505411) can simply be interpreted as the ICC because the total variance adds up to 1 (the slight deviation in the fourth decimal is due to the fact that $ZVelocity$ is not perfectly normally distributed).

Note that the residual variance (denoted as σ_e^2 in the equation to obtain the ICC and estimated at 0.494) represents the variation observed within each neuron, i.e., the variability in velocity measures taken from the same neuron.

The statistical significance of the variation in the intercept can also be assed. However, the Wald test reported in the table is not appropriate to test significance of variances (i.e., the asymptotic Wald test assumes normally distributed variance components, which is unrealistic [2]). Whether the variance component is significantly different from 0 can, however, be tested using a chi-square (χ^2) test. If we square the Wald Z statistic in the table, we get approximately a chi-square value, with the number of degrees of freedom being 1 (i.e., we test only 1 parameter,

Table S3 Syntax and selected output for model including fixed effect of *Location* through SPSS MIXED

```
MIXED
ZVelocity with int Location
/fixed int Location | noint
/random int | subject(Neuron_ID) covtype(un)
/METHOD = ML
/print solution testcov r.
```

Estimates of Fixed Effects[a]

Parameter	Estimate	Std. Error	df	t	Sig.	95% Confidence Interval Lower Bound	95% Confidence Interval Upper Bound
int	.282169	.160356	20.328	1.760	.094	-.051981	.616319
Location	-.564338	.028874	1980.209	-19.545	.000	-.620965	-.507710

a. Dependent Variable: Zscore(Velocity).

Covariance Parameters

Estimates of Covariance Parameters[a]

Parameter		Estimate	Std. Error	Wald Z	Sig.	95% Confidence Interval Lower Bound	95% Confidence Interval Upper Bound
Residual		.414184	.013164	31.464	.000	.389171	.440805
int [subject = Neuron_ID]	Variance	.505968	.161311	3.137	.002	.270860	.945148

a. Dependent Variable: Zscore(Velocity).

namely the variance of the intercept)[1] . So we get:

$$\chi^2(1) = (3.132)^2 = 9.809. \tag{2}$$

Since a variance component cannot be negative and this parameter is thus subject to boundary constraints (see e.g. [3–5]), the accompanying p-value, which equals .002, needs to be divided by 2: $p = .001$. Assuming $\alpha = .05$, this test is significant, i.e., the variation of the intercept over neurons is significantly different from 0.
Note that in research design B, not accommodating the variation in the intercept results in a decreased power to detect the overall experimental effect (which is different to research design A, where not accommodating the variation in the intercept results in an inflated false positive rate).

Model including fixed effect of Location on vesicle velocity After we estimated the intercept only model and the ICC, we add Location to our model to identify its effect on vesicle velocity, i.e., is the velocity of vesicles different in axons compared to dendrites. We first add Location only as a fixed variable to the model, and then extend the model to include the possible variation in the difference between axonal and dendritic measurements over neurons. The syntax and selected output for the model only including the fixed effect of location is presented in Table S3.
We see that the overall effect of location equals -0.564. However, we cannot draw any conclusions on the significance of this effect, as we did not accommodate the

[1]Note that SPSS prints -2 Log Likelihood information in the table with information criteria. Usually, this -2LL information is used to calculate the chi-square test. However, SPSS sometimes uses pseudo maximum likelihood estimation, and then the -2LL values of different models cannot be used to compute a chi-square value.

possible variance of the effect of location over neurons. Note that adding the experimental variable Location results in a decreased residual error (i.e., from .494 to .414), i.e., *ZLocation* partly explains why the observations *within* neurons vary. Also note that the variation between neurons (i.e., the intercept variance of 0.506) remains almost the same (up to the third decimal place) compared to the model that does not include *ZLocation*. However, the interpretation of the variation in the intercept is now changed to neuron-related variation in the mean velocity of axonal measurements specifically (while it was interpreted as neuron-related variation in mean velocity in general (i.e., both axonal and dendritic measures) before Location was included as predictor in the model).

Model including both the fixed and random effect of Location on vesicle velocity
The syntax and selected output for the model including variance in the effect of Location is presented in Table S4. Note that we save some variables in the last line of the syntax, these are used later to plot the results. Also note that we set `covtype` to `DIAG`, i.e., the variance-covariance matrix between the parameter estimates is diagonal, meaning that we assume that the intercept and Location effect parameters have variances (on the diagonal) but that they do not correlate (i.e., the covariance, which is noted on the off-diagonal elements, is 0, i..e, the neuron-specific mean axonal velocity is not related to the neuron-specific effect of *ZLocation* on vesicle velocity).

The overall effect of *Location* on vesicle velocity is approximately the same as in the previous analysis: -0.565, and is highly significant with $p < .001$. The effect size d of *Location* is obtained through γ_{10}/σ_e^2 [6], which corresponds to $-0.565/0.387 = -1.460$. By convention, effect sizes of 0.20, 0.50 and 0.80 are considered small, medium, and large, respectively [7]. As such, the overall effect of location

Table S4 Syntax and selected output for model including fixed and random effect of *Location* **through SPSS MIXED**

```
MIXED
ZVelocity with int Location
/fixed int Location | noint
/random int Location | subject(Neuron_ID) covtype(un)
/METHOD = ML
/print solution testcov r
/SAVE = FIXPRED SEFIXP PRED SEPRED RESID.
```

Estimates of Fixed Effects[a]

Parameter	Estimate	Std. Error	df	t	Sig.	95% Confidence Interval Lower Bound	95% Confidence Interval Upper Bound
int	.280619	.147792	20.024	1.899	.072	-.027647	.588885
Location	-.564668	.079145	20.050	-7.135	.000	-.729735	-.399601

a. Dependent Variable: Zscore(Velocity).

Covariance Parameters

Estimates of Covariance Parameters[a]

Parameter		Estimate	Std. Error	Wald Z	Sig.	95% Confidence Interval Lower Bound	95% Confidence Interval Upper Bound
Residual		.387053	.012362	31.310	.000	.363567	.412056
int + Location [subject = Neuron_ID]	Var: int	.429064	.138063	3.108	.002	.228363	.806156
	Var: Location	.109696	.039569	2.772	.006	.054094	.222449

a. Dependent Variable: Zscore(Velocity).

corresponds to a (very) large effect. The 95% confidence interval (CI) assuming a normal distribution is obtained through $\gamma_{10} \pm Z_{1-\alpha} * SE_{\gamma_{10}}$, which corresponds to -0.565 ± 0.079 * 1.96 = [-0.720, -0.410] (the deviation with the SPSS output is because SPSS uses the t distribution with 20.05 degrees of freedom to obtain the 95% CI).

The effect of *Location* on vesicle velocity, however, varies over neruons with a medium sized variation: the neuron-related variance of the experimental effect equals 0.110. To interpret the variation in the effect of Location over neurons, consider the following. The variance of 0.110 corresponds to a standard deviation of

Table S5 Syntax and selected output for intercept-only model through SPSS MIXED

```
GGRAPH
 /GRAPHDATASET NAME="graphdataset"
VARIABLES= Location PRED_1 Neuron_ID
MISSING=LISTWISE REPORTMISSING=NO
 /GRAPHSPEC SOURCE=INLINE.
BEGIN GPL
 SOURCE: s=userSource(id("graphdataset"))
 DATA: Location =col(source(s), name("Location"))
 DATA: PRED_1=col(source(s), name("PRED_1"))
 DATA: Neuron_ID =col(source(s), name("Neuron_ID"), unit.category())
 GUIDE: axis(dim(1), label("Location"))
 GUIDE: axis(dim(2), label("Predicted Values"))
 GUIDE: legend(aesthetic(aesthetic.color.interior), label("Neuron_ID"))
 SCALE: cat(aesthetic(aesthetic.color.interior), include("0", "1", "2", "3",
"4", "5", "6", "7", "8", "9", "10", "11", "12", "13", "14", "15", "16", "17",
"18", "19"))
 ELEMENT: line(position(Location*PRED_1), color.interior(Neuron_ID),
missing.wings())
END GPL.
```

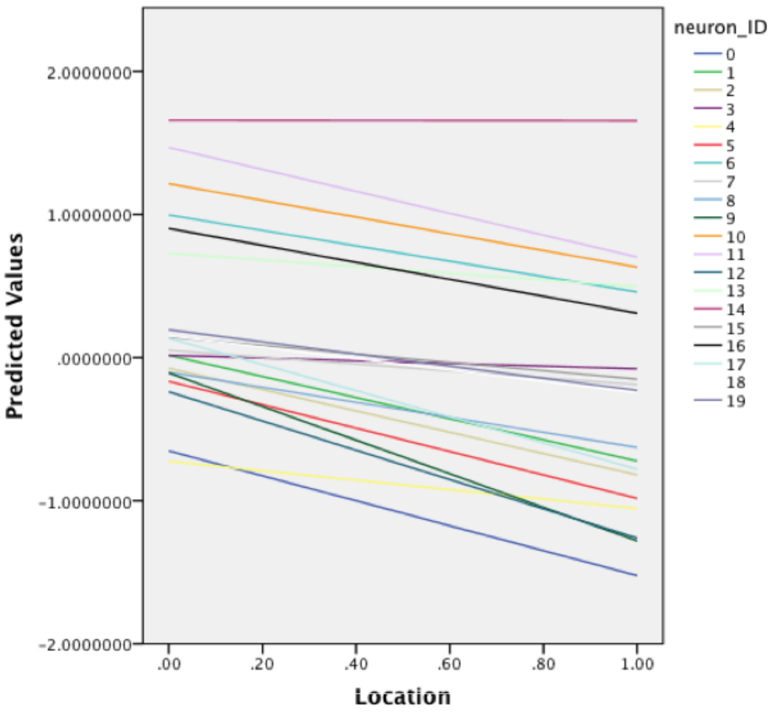

0.332. Assuming normality of the cluster specific deviations from the overall effect, β_{1j}, about 95% of the neuron-individual Location effects would be between -0.565 - 1.96 * 0.332 = -1.216 and -0.565 + 1.96 * 0.332 = 0.086; virtually all comparisons between velocity of axonal and dendritic vesicles show a negative effect of location, i.e., lower velocity in dendritic vesicles. The percentage of effects showing a positive effect of Location (again assuming normality) is about 4%. We can also plot the neuron-specific effects of Location on vesicle velocity, syntax and selected output is presented in Table S5. This plot clearly shows that the velocity is usually lower in dendrites than in axons, but that the extent to which it is lower is not the same for every neuron.

As simulations presented in Fig. 4 in the main text showed that the Type I error rate can already be much increased when the cluster-related variation in the experimental effect equals 0.025, multilevel analysis is certainly advised when the neuron-related variation in the experimental effect equals 0.11.

To test if the variation in the experimental effect over neurons is statistically significant, we again use the chi-square (χ^2) test appropriate for variance components, resulting in:

$$\chi^2(1) = (2.772)^2 = 7.684. \tag{3}$$

Dividing the accompanying p-value by 2 results in $p = .003$. Assuming $\alpha = .05$, this test is significant, i.e., the neuron-related variation in the effect of Location on velocity is significantly different from 0, i.e., the effect of Location on velocity varies over neurons. Note that the neuron-related variation in the effect of Location both partly explains why measurements within an axonal or dendritic location vary within a neuron (i.e., the residual variance) and why the mean velocity of axonal measurements varies over neurons (i.e., the intercept variance): both variance components are now decreased compared to the previous model.

In summary, based on the multilevel analyses of these data one would conclude that:

- The intercept shows variation across neurons (i.e., the mean velocity of axonal measurements varies over neurons)
- Vesicle velocity differs between axonal and dendritic measurements, where the velocity of dendritic vesicles is slower than those of axonal vesicles, but
- The degree of difference between axonal and dendritic vesicle velocity varies across neurons.

Table S6 Corresponding R code

```
# Load SPSS datafile in R
library(foreign)
data.velocity <- read.spss(file = "Velocity axon-dendrite.sav", use.value.labels = FALSE, to.data.frame = TRUE)
head(data.velocity)

# Scatterplot of standardized velocity by Neuron_ID, axonal and dendritc measurements color-coded
attach(data.velocity)
plot(y = ZVelocity, x = Neuron_ID, type = "n", las = 1, ylab = "Standardized value of Velocity")
points(y = ZVelocity[Location == 0], x = Neuron_ID[Location == 0], col = "royalblue")
points(y = ZVelocity[Location == 1], x = Neuron_ID[Location == 1]+.1, col = "springgreen3")
legend("topleft", pch = 1, col = c("royalblue", "springgreen3"), legend = c("axonal measurements", "dendritic measurements"), bty = "n")

# Intercept only model
library(lme4)
ML1 <- lmer(ZVelocity ~ (1 | Neuron_ID), data.velocity, REML = FALSE)
ML1

# Model with fixed effect for Location
ML2 <- lmer(ZVelocity ~ Location + (1 | Neuron_ID), data.velocity, REML = FALSE)
ML2

# Model with both fixed and random effect of Location
ML3 <- lmer(ZVelocity ~ Location + (Location | Neuron_ID), data.velocity, REML = FALSE)
ML3

# Plotting Neuron specific effects of Location on vesicle velocity
pred.velocity <- fitted(ML3)
pred.velocity.aggr <- aggregate(pred.velocity, by = list(Location = Location, Neuron_ID = Neuron_ID), FUN = mean)
col.neurons <- rainbow(20)
plot(y = pred.velocity.aggr$x, x = pred.velocity.aggr$Location, ylab = "Predicted standardized velocity", xlab = "Location measured vesicle", xaxt = "n", las = 1, type = "n", ylim = c(-1.5, 3))
axis(side = 1, at = c(0,1), labels = c("Axonal", "Dendritic"))
for(i in 1:20){
points(y = pred.velocity.aggr$x[pred.velocity.aggr$Neuron_ID == (i-1)], x = pred.velocity.aggr$Location[pred.velocity.aggr$Neuron_ID == (i-1)], col = col.neurons[i], type = "b", ylim = c(-1.5, 3))
}
legend("topleft", legend = paste("Neuron", 0:19), col = col.neurons, lty = 1, bty = "n", ncol = 4)
```

References

[1] Raudenbush, S.W., Liu, X.: Statistical power and optimal design for multisite randomized trials. Psychological methods **5**(2), 199–213 (2000)

[2] Bryk, A.S.a.W.R.: Hierarchical Linear Models: Applicationsand Data Analysis Methods. Sage, Newbury Park, CA (1992)

[3] Dominicus, A., Skrondal, A., Gjessing, H.K., Pedersen, N.L., Palmgren, J.: Likelihood ratio tests in behavioral genetics: problems and solutions. Behavior genetics **36**(2), 331–340 (2006)

[4] Berkhof, J., Snijders, T.A.: Variance component testing in multilevel models. Journal of Educational and Behavioral Statistics **26**(2), 133–152 (2001)

[5] Stoel, R.D., Garre, F.G., Dolan, C., Van Den Wittenboer, G.: On the likelihood ratio test in structural equation modeling when parameters are subject to

boundary constraints. Psychological Methods **11**(4), 439 (2006)

[6] Hedges, L.V.: Effect sizes in cluster-randomized designs. Journal of Educational and Behavioral Statistics **32**(4), 341–370 (2007)

[7] Cohen, J.: Statistical Power Analysis for the Behavioral Sciences, 2nd edn. Erlbaum, Hillsdale, NJ (1988)
