# Supplementary material for: Multilevel analysis quantifies variation in the experimental effect while optimizing power and preventing false positives
Source: BMC Neurosci. 2015 Dec 19;16:94. doi: 10.1186/s12868-015-0228-5 (PMC4684932; doi:10.1186/s12868-015-0228-5)
Supplement: Supplementary file 2 — 10.1186/s12868-015-0228-5 Calculating the optimal allocation of sample sizes and estimating statistical power to detect the overall experimental effect. Explanation on how to calculate the optimal allocation of sample sizes over clusters and within clusters given the available resources, and explanation on how to estimate power for a balanced (i.e., the number of observations per condition are equal both between conditions and between clusters) 2-level multilevel model without covariates. [file 12868_2015_228_MOESM2_ESM.pdf]

Additional file 2

Calculating the optimal allocation of sample sizes and estimating statistical power to detect the overall experimental effect

Calculating the optimal allocation of sample sizes

As shown in Fig. 5 in the main text, power increases more when extra clusters are added, compared to the increase yielded when extra observations per cluster are added. However, the costs of including an additional cluster (C_2) are usually higher than including an additional observation within each cluster (C_1). The optimal balance between the number of clusters (N) and observations per cluster (n) in terms of power and costs can be determined given the cost ratio between C_1 and C_2, the estimated (or expected) variation of the experimental effect over clusters, and the total amount of available resources. First, one estimates the optimal number of observations per cluster $n_{optimal}$ by:

$$n_{optimal} = 2 * \sqrt{\frac{C_2}{C_1 * \sigma^2_{u1}}}, \tag{1}$$

where σ^2_{u1} is the standardized variance of the experimental effect over clusters. The magnitude of σ^2_{u1} can be interpreted according to the guidelines of Raudenbush and Liu [1], i.e., values of σ^2_{u1} equaling 0.05, 0.10, and 0.15 are considered small, medium, and large, respectively. The standardized variance of the experimental effect is obtained when the data has the following structure. The outcome variable is standardized such that $\sim \mathcal{N}(0,1)$ (i.e., the variable is transformed such that it has a mean of 0 and standard deviation of 1) and the dummy indicator of the experimental condition X is coded either as 0 and 1, or, if one wants to center the experimental variable, as -0.5 and 0.5 (note that the cluster-related variation in the intercept σ^2_{u0} is not part of equation 1. As the estimated standard error of the overall experimental effect γ_{10} is not influenced by the cluster-related variation in the intercept, σ^2_{u0} does not influence the optimal number of observations per cluster).

The total costs of a study T are

$$T = N(C_1 * n + C_2). \tag{2}$$

Therefore, the number of clusters N for $n_{optimal}$ can be obtained by

$$N \leq \frac{T}{n_{optimal} * C_1 + C_2}. \tag{3}$$

To illustrate calculating the optimal allocation of sample sizes, say that we have 4,000 monetary units to spend on a study of differences between axons and dendrites with respect to a specific characteristic of the cell. It costs 80 units to plate a cell, and 1 unit to obtain an observation from either an axon or dendrite within a cell. From previous studies we know that the standardized variance of the experimental effect over clusters is approximately 0.10 and we set $\sigma^2_{u1} = 0.10$ accordingly. When one does not poses any a priori information on the expected variation in

the experimental effect, one can choose to calculate the optimal distribution of resources/observations for different values of the cluster-related variation in the experimental effect, using the guidelines of Raudenbusch and Lui [1].
The resulting $n_{optimal} = 2 * \sqrt{80/(1 * 0.05)} = 56.6$; rounding down to the nearest integer gives an optimal number of 28 dendritic observations and 28 axonal observations per cell (note that rounding *down* is recommended for $n_{optimal}$. This leaves more resources to spend on number of clusters: more clusters is always more beneficial than more observations per cluster in terms of power). The corresponding number of clusters N = 4,000/ (56 * 1 + 80) = 29 (note that we are rounding *down* here as well, since rounding up results in surpassing the budget). In summary, given the amount of cluster-related variation in the experimental effect, cost ratio, and available resources, the optimal balance between power and costs is to plate 29 cells from each of which we collect 28 dendritic observations and 28 axonal observations. Important to note is that the optimal allocation of observations does *not* guarantee sufficient power to detect the experimental effect of interest. Specifically, optimal allocation of samples only maximizes power given the available resources and the expected variation of the experimental effect over clusters σ^2_{u1}. Therefore, it is advised to estimate the expected power with the obtained $n_{optimal}$ and N, given specific values of the effect size d of the overall experimental effect γ_{10}, α-level and the variance components (i.e., residual error σ^2_e and the standardized variance of the experimental effect over clusters σ^2_{u1}). How this is done, is explained in the next section.

Estimating the statistical power to detect the overall experimental effect
The power for a balanced (i.e., the number of observations per condition is both equal between conditions and over cluster) 2-level multilevel model without covariates is estimated as follows. In research design B, the significance of the overall experimental effect γ_{10} can be tested using an F-test. Here, F follows a noncentral F-distribution with degrees of freedom 1 and N-1, and the noncentrality parameter λ, $F(1, N-1; \lambda)$, where N denotes the number of clusters[1].
The estimated power can therefore be obtained as follows. First calculate the noncentrality parameter λ. Next, use the obtained value λ and the degrees of freedom to obtain the probability of exceeding the critical value for F in the noncentral F-distribution (F_{crit}). The noncentrality parameter λ is given by (adjusted notation from equation 15 Raudenbusch and Liu [1]):

$$\lambda = \frac{n * N * \gamma^2_{10}}{n * \sigma^2_{u1} + 4\sigma^2_e}. \quad (4)$$

[1] Testing significance of the overall experimental effect using the noncentral F test with 1 and N -1 degrees of freedom is approximately similar to testing the overall experimental effect using a t-distribution with degrees of freedom N-1-(*number experimental variables*) put forward by Bryk and Raudenbush [2]. The latter is also used in the multilevel analysis package HLM [3]. Most statistical packages, however, use the Wald test [4] to assess the statistical significance of the overall experimental effect. In the Wald test, Z is evaluated against the standard normal distribution, where Z is obtained by Z = (*overall experimental effect*) / (*standard error of overall experimental effect*). As the standard normal distribution does not depend on degrees of freedom, sample size is not taken into account in evaluating the significance of the overall experimental effect. When the number of clusters is small, the difference in the obtained significance value for the Wald test and the noncentral F or t test becomes considerable, and using the noncentral F or t test is conservative.

When λ is obtained, the probability to exceed the critical value of F in the noncentral F distribution F_{crit} is estimated by:

$$Prob[F(1, N-1; \lambda) > F_{crit} = 1 - Prob[F(1, N-1; \lambda) < F_{crit}. \tag{5}$$

The probability of the last term in equation 5, $Prob[F(1, N-1; \lambda) < F_{crit}$, can easily be obtained in widely used statistical packages like SAS and R, and in online calculators. Hence, when the noncentrality parameter λ and the degrees of freedom are known, the estimated power is relatively easily obtained.
A difficulty with obtaining λ is that the effect size and variance components are usually not known beforehand. The solution is to assume a standardized model such that 1) the degree of variation of the experimental effect over clusters σ^2_{u1} can be chosen according to the rules of Raudenbush and Liu [1] where 0.05, 0.10 and 0.15 are considered small, medium and large measures of σ^2_{u1}, respectively, 2) the magnitude of the experimental effect γ_{10} can be chosen according to the conventions for effect size d, where a standardized effect of 0.20, 0.50 and 0.80 are considered small, medium and large [5], and 3) the residual error σ^2_e can be set to 1. Now, based on previous studies, one can make an educated guess and/or consider a range of values to acquire a feeling for the obtained power for the planned research under various, more and less advantageous, conditions.
We illustrate obtaining the estimated statistical power by continuing the previous example. When we assume a small overall experimental effect $\gamma_{10} = 0.20$, filling in the parameters of the model ($n_{optimal} = 56$ observations per cell, $N = 29$ cells, variance in the experimental effect $\sigma^2_{u1} = 0.10$ and $\sigma^2_e = 1.00$) results in

$$\lambda = \frac{56 * 29 * 0.20^2}{56 * 0.10 + 4 * 1}. \tag{6}$$

Next, using the statistical package R [6], we obtain F_{crit} with the quantile density function for the F distribution, `qf()`:

```
Fcrit <- qf(1-alpha, 1, N-1),
```

where `alpha` is the chosen significance level. For $\alpha = 0.05$ and $N = 29$, F_{crit} equals 4.20. Next, the calculated values for λ and F_{crit} are used to obtain the probability of exceeding the critical value for F in the noncentral F distribution. We do this by using the distribution function of the F distribution in R, `pf()`:

```
power <- 1- pf(Fcrit, 1, N-1, lambda),
```

where `lambda` is the noncentrality parameter λ. The estimated power equals 71%.
If the number of observations vary per condition and/or vary over clusters (i.e., an unbalanced desing), the mean cluster size may be used instead. The equations will then give an approximation, and deviations from a balanced design generally result in decreased power.
The program Power in Two-level designs (PinT; http://www.stats.ox.ac.uk/~snijders/, based on [7]]) can be used to obtain estimates for power in more complex cases (e.g., unbalanced designs, or designs with covariates at the observational or cluster level).

References

[1] Raudenbush, S.W., Liu, X.: Statistical power and optimal design for multisite randomized trials. Psychological methods **5**(2), 199–213 (2000)

[2] Bryk, A.S.a.W.R.: Hierarchical Linear Models: Applicationsand Data Analysis Methods. Sage, Newbury Park, CA (1992)

[3] Raudenbush, S.W., Yang, M.-L., Yosef, M.: Maximum likelihood for generalized linear models with nested random effects via high-order, multivariate laplace approximation. Journal of computational and Graphical Statistics **9**(1), 141–157 (2000)

[4] Wald, A.: Tests of statistical hypotheses concerning several parameters when the number of observations is large. Transactions of the American Mathematical society **54**(3), 426–482 (1943)

[5] Cohen, J.: Statistical Power Analysis for the Behavioral Sciences, 2nd edn. Erlbaum, Hillsdale, NJ (1988)

[6] R Core Team: R: A Language and Environment for Statistical Computing. R Foundation for Statistical Computing, Vienna, Austria (2013)

[7] Snijders, T.A., Bosker, R.J.: Standard errors and sample sizes for two-level research. Journal of Educational and Behavioral Statistics **18**(3), 237–259 (1993)
